# 8-Epixanthatin Suppresses RANKL-Induced Osteoclast Differentiation via Inhibition of NF-κB and MAPK Signaling

**DOI:** 10.3390/ijms27083578

**Published:** 2026-04-17

**Authors:** Lifang Zhang, Vishwa Deepak

**Affiliations:** 1 Osteoimmunology and Drug Discovery Research Group, Department of Biology, College of Science, Mathematics and Technology, Wenzhou-Kean University, 88 Daxue Road, Wenzhou 325060, China; 2 Dorothy and George Hennings College of Science, Mathematics and Technology, Kean University, 1000 Morris Ave, Union, NJ 07083, USA; 3 International Frontier Interdisciplinary Research Institute (IFIRI), Wenzhou-Kean University, Wenzhou 325060, China; 4 Wenzhou Municipal Key Laboratory for Applied Biomedical and Biopharmaceutical Informatics, Wenzhou-Kean University, Ouhai, Wenzhou 325060, China; 5 Zhejiang Bioinformatics International Science and Technology Cooperation Center, Wenzhou-Kean University, Ouhai, Wenzhou 325060, China; 6 Zhejiang-Malaysia Joint Laboratory for Rare Medicinal Resources, Wenzhou-Kean University, 88 Daxue Road, Ouhai, Wenzhou 325060, China

**Keywords:** osteoclast differentiation, receptor activator of nuclear factor kappa-B ligand (RANKL), nuclear factor κB (NF-κB), mitogen-activated protein kinase (MAPK), osteoporosis, bone resorption, natural compounds

## Abstract

Osteoclast hyperactivity represents a central mechanism in pathological bone destruction, underscoring the importance of discovering novel anti-resorptive compounds. In this study, we present early-stage evidence that 8-Epixanthatin can inhibit osteoclast differentiation induced by receptor activator of nuclear factor kappa-B ligand (RANKL). 8-Epixanthatin exhibited no significant cytotoxicity at the concentrations used for osteoclast differentiation studies. The compound showed concentration-dependent reductions in TRAP-positive multinucleated osteoclasts, with an IC_50_ value of 2.3 μM. Our mechanistic investigations revealed that 8-Epixanthatin interferes with RANKL-activated signaling networks, particularly nuclear factor kappa-B (NF-κB) and mitogen-activated protein kinase (MAPK) cascades. Collectively, these observations identify 8-Epixanthatin as a promising lead structure for anti-osteoclast drug discovery.

## 1. Introduction

Bone tissue maintains equilibrium through balanced osteoblast-mediated formation and osteoclast-driven resorption [1]. Pathological conditions including osteoporosis, inflammatory osteolysis, and tumor-associated bone destruction arise when this balance tilts toward excessive resorption [2]. Receptor activator of nuclear factor kappa-B ligand (RANKL) is the principal cytokine driving osteoclast differentiation. Upon RANKL–RANK engagement, tumor necrosis factor (TNF) receptor-associated factor 6 (TRAF6) is recruited, leading to activation of downstream signaling pathways. Specifically, TRAF6 activates the inhibitor of kappa-B kinase (IKK) complex, resulting in IκB phosphorylation and subsequent nuclear translocation of NF-κB p65. Concurrently, TRAF6 activates transforming growth factor-β-activated kinase 1 (TAK1), which triggers mitogen-activated protein kinase (MAPK) cascades including extracellular signal-regulated kinase 1/2 (ERK1/2), c-Jun N-terminal kinase (JNK), and p38 [3,4,5]. These signaling pathways collectively induce the expression of osteoclast-specific genes such as nuclear factor of activated T cells cytoplasmic 1 (*NFATc1*), c-Fos, tartrate-resistant acid phosphatase (*TRAP*), and cathepsin K (*Ctsk*) [6]. 

Current anti-resorptive therapies such as bisphosphonates and denosumab effectively reduce bone loss but are associated with adverse effects during prolonged use, including osteonecrosis of the jaw and atypical femoral fractures [7]. These limitations have intensified interest in safer therapeutic alternatives, particularly small-molecule inhibitors derived from natural products.

8-Epixanthatin (8-E), a sesquiterpene lactone derived from *Xanthium* species, has been reported to exhibit diverse biological activities, including anti-angiogenic, antiviral, and anti-inflammatory properties [8]. Structurally, sesquiterpene lactones contain α,β-unsaturated carbonyl groups that undergo Michael addition with nucleophilic cysteines in target proteins, enabling covalent modulation of redox-sensitive signaling pathways [9]. This electrophilic reactivity has been demonstrated for structurally analogous sesquiterpene lactones: parthenolide alkylates Cys179 of IKKβ [10], and helenalin covalently modifies Cys38 of NF-κB p65 [11], shown using biochemical and chemical approaches. Given that NF-κB and MAPK signaling pathways involve redox-sensitive cysteine residues and are essential for osteoclast differentiation, we hypothesized that 8-E may suppress osteoclastogenesis through similar mechanisms. Despite its pharmacological relevance, 8-E’s effects on osteoclast biology remain uncharacterized. This study presents initial evidence that 8-E inhibits RANKL-induced osteoclast differentiation by disrupting critical downstream signaling pathways.

## 2. Results

### 2.1. 8-Epixanthatin Exhibits Minimal Cytotoxicity in Osteoclast Precursor Cells

**Figure 1:**
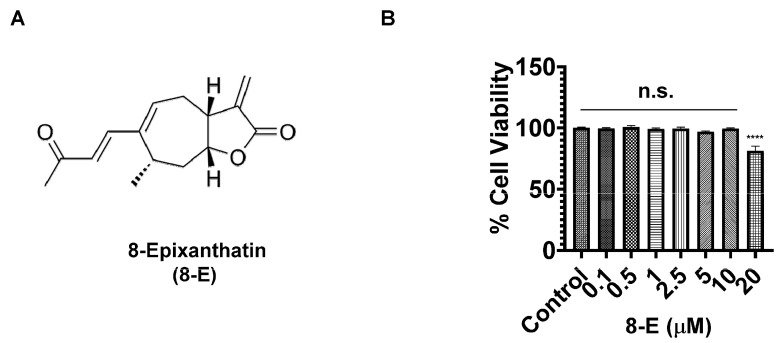
Structure and cytotoxicity profile of 8-Epixanthatin (8-E). (**A**) Chemical structure of 8-Epixanthatin showing the characteristic α,β-unsaturated carbonyl moiety of sesquiterpene lactones. (**B**) RAW264.7 cells were treated with indicated concentrations of 8-Epixanthatin (0.1–20 μM) for 48 h, and cell viability was assessed by CCK-8 assay. Cell viability remained >99% at concentrations ≤10 μM. Data are presented as mean ± SD from three independent experiments. Statistical significance was determined by one-way ANOVA followed by Tukey’s multiple comparisons test (**** *p* < 0.0001; n.s., not significant).

The chemical structure of 8-Epixanthatin (8-E), featuring the characteristic α,β-unsaturated carbonyl moiety of sesquiterpene lactones, is shown in Figure 1A. RAW264.7 cells were treated with increasing concentrations of 8-E (0.1–20 μM) for 48 h. Cell viability remained ~99% at concentrations up to 10 μM, with a modest reduction to approximately 81% observed only at 20 μM (Figure 1B). These results indicated that concentrations up to 10 μM could be used for subsequent experiments without confounding effects of cytotoxicity.

### 2.2. 8-Epixanthatin Potently Inhibits RANKL-Induced Osteoclastogenesis

**Figure 2:**
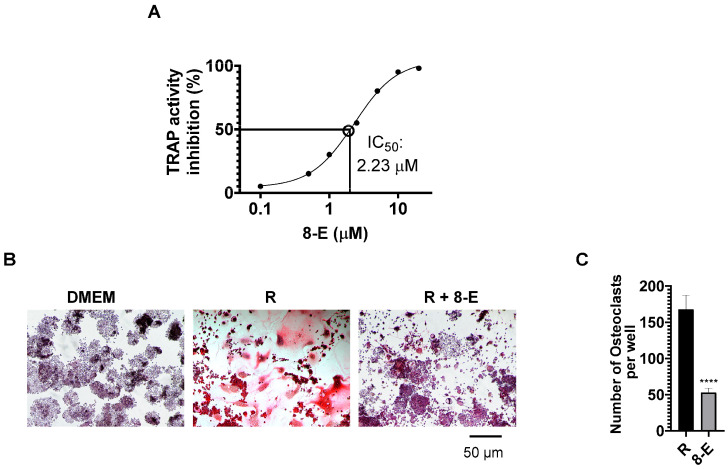
8-E potently inhibits RANKL-induced osteoclastogenesis in a dose-dependent manner. (**A**) Dose-response curve showing inhibition of osteoclast formation by 8-E. RAW264.7 cells were stimulated with RANKL (30 ng/mL) in the presence of increasing concentrations of 8-E (0.1–10 μM) for 5 days. Cells were assayed for TRAP activity (IC_50_ = 2.3 μM). Data are mean ± SD (*n* = 3 independent experiments). (**B**) Representative images of TRAP-stained cultures showing osteoclast formation. Left panel: Control; Middle panel: RANKL (30 ng/mL) stimulation showing numerous large, multinucleated TRAP-positive osteoclasts (dark pink/red cells); Right panel: RANKL + 8-E (2.5 μM) showing markedly reduced osteoclast number and size. Scale bar = 50 μm. Original magnification 20×. (**C**) Quantification of TRAP-positive multinucleated osteoclasts (≥3 nuclei) per well from cultures shown in panel B. Data are mean ± SD from three independent experiments. **** *p* < 0.0001 compared to RANKL alone by unpaired Student’s *t*-test.

To evaluate the effect of 8-E on osteoclast differentiation, RAW264.7 cells were stimulated with receptor activator of nuclear factor kappa-B ligand (RANKL) in the presence of varying concentrations of the compound (0.1–10 μM). Dose-response analysis revealed concentration-dependent suppression of tartrate-resistant acid phosphatase (TRAP) activity (Figure 2A). The compound exhibited potent anti-osteoclastogenic activity with an IC_50_ of 2.3 μM, indicating that 8-E inhibits osteoclast formation in the low micromolar range. Representative TRAP staining images demonstrated abundant large, multinucleated TRAP-positive osteoclasts (appearing as dark purple cells) in RANKL-stimulated cultures, whereas treatment with 2.5 μM 8-E markedly reduced both osteoclast number and size (Figure 2B). Vehicle (DMEM) control cultures showed only scattered mononuclear cells with very few osteoclasts formed. Quantitative analysis confirmed a significant reduction in the number of TRAP-positive multinucleated cells (≥3 nuclei) from 168 ± 19 osteoclasts per well in RANKL-treated cultures to 53 ± 6 osteoclasts per well following treatment with 2.5 μM 8-E (*p* < 0.0001, representing approximately 69% inhibition) (Figure 2C). These findings demonstrate that 8-E potently inhibits RANKL-induced osteoclast differentiation at concentrations well below those causing cytotoxicity.

### 2.3. 8-Epixanthatin Suppresses RANKL-Induced NF-κB and MAPK Signaling and Inhibits Expression of Osteoclast Marker Genes

**Figure 3:**
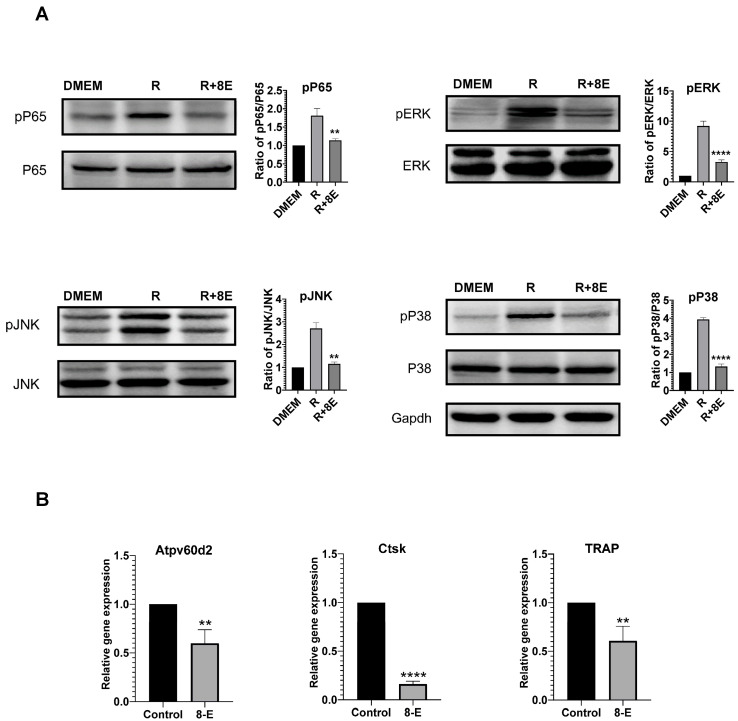
8-E suppresses RANKL-induced activation of NF-κB and MAPK signaling pathways and inhibits expression of osteoclast marker genes. (**A**) RAW264.7 cells were pretreated with 8-E (10 μM, 8E) or vehicle (DMSO) for 2 h, then stimulated with RANKL (50 ng/mL) for 15 min. DMEM = unstimulated control. Cell lysates were analyzed by Western blot for phosphorylated and total levels of p65, ERK1/2, JNK, and p38. GAPDH served as loading control. Representative Western blots are shown with corresponding densitometric quantification in bar graphs. Phosphorylated protein levels were normalized to corresponding total protein levels. (**B**) RAW264.7 cells were treated with RANKL (30 ng/mL) in the presence or absence of 8-E (2.5 μM) for 5 days. Expression of osteoclast marker genes *Atp6v0d2*, *cathepsin K* (*Ctsk*), and *TRAP* was analyzed by quantitative RT-PCR. Data are mean ± SD from three independent experiments. ** *p* < 0.01, **** *p* < 0.0001 compared to RANKL alone. Statistical significance was determined by one-way ANOVA followed by Tukey’s multiple comparisons test.

To elucidate the molecular mechanisms underlying 8-E’s anti-osteoclastogenic effect, we examined RANKL-activated signaling pathways. RANKL stimulation induced robust phosphorylation of receptor activator of nuclear factor kappa-B ligand (NF-κB) p65 at Ser536, which was reduced by approximately 30% following pretreatment with 10 μM 8-E, while total p65 levels remained unchanged (Figure 3A). Similarly, 8-E substantially suppressed RANKL-induced phosphorylation of all three mitogen-activated protein kinase (MAPK) family members: extracellular signal-regulated kinase 1/2 (ERK1/2) (~70% reduction), c-Jun N-terminal kinase (JNK) (~48% reduction), and p38 (~63% reduction), without affecting total kinase expression (Figure 3A).

To confirm transcriptional suppression, we examined expression of osteoclast marker genes. 8-E (2.5 μM) significantly reduced RANKL-induced expression of *Atp6v0d2* (~40%, *p* < 0.01), *cathepsin K* (*Ctsk*) (~85%, *p* < 0.0001), and *tartrate-resistant acid phosphatase* (*TRAP*) (~40%, *p* < 0.01) (Figure 3B). These results demonstrate that 8-E targets multiple RANKL-responsive signaling cascades and downstream gene expression essential for osteoclast differentiation.

## 3. Discussion

This study provides preliminary evidence that 8-Epixanthatin (8-E), a natural sesquiterpene lactone derived from *Xanthium* species, potently inhibits osteoclast differentiation by targeting key receptor activator of nuclear factor kappa-B ligand (RANKL)-responsive signaling pathways. The compound exhibited an IC_50_ of 2.3 μM for suppression of osteoclastogenesis, which lies within a therapeutically relevant range and compares favorably with other natural product-derived osteoclast inhibitors reported in the literature. Importantly, this anti-osteoclastogenic activity was achieved without cytotoxicity at effective concentrations, supporting the potential of 8-E as a lead compound for anti-resorptive drug development.

**Figure 4:**
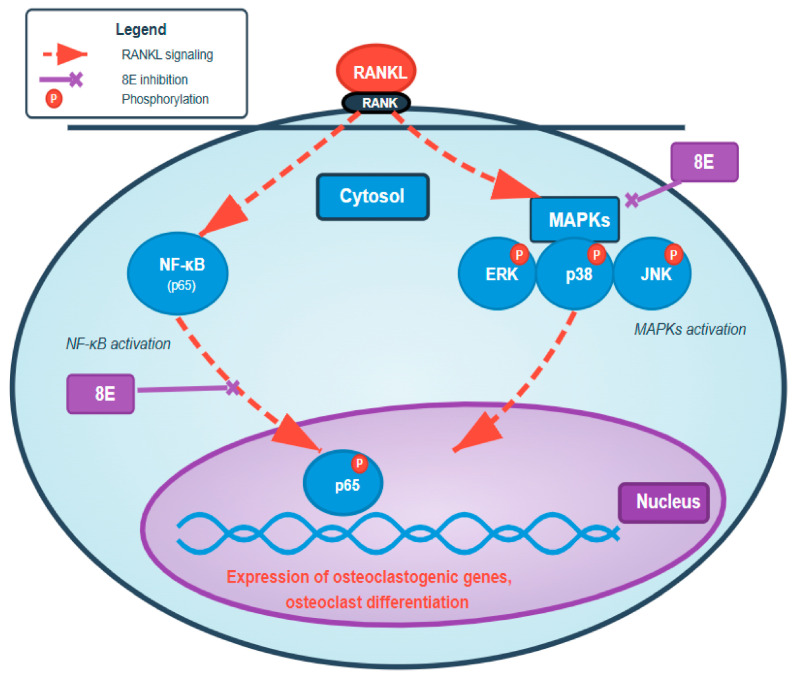
Proposed mechanism of 8-E inhibition of RANKL-induced osteoclastogenesis. Schematic illustrating that 8-E suppresses RANKL-induced osteoclast differentiation, at least in part, by inhibiting activation of NF-κB and MAPK (ERK, JNK, p38) signaling pathways downstream of RANK. Attenuation of these pathways reduces the expression of osteoclastogenic genes required for osteoclast differentiation and function.

The inhibition of both nuclear factor kappa-B ligand (NF-κB) and mitogen-activated protein kinase (MAPK) pathways by 8-E is mechanistically significant, as these pathways function cooperatively to drive osteoclast differentiation. NF-κB activation is required for the initial induction of nuclear factor of activated T cells cytoplasmic 1 (NFATc1), the master regulator of osteoclastogenesis. MAPK signaling particularly through extracellular signal-regulated kinase 1/2 (ERK1/2) and c-Jun N-terminal kinase (JNK) regulates induction of c-Fos and activator protein 1 (AP-1) activity. These pathways cooperate with NFATc1 to drive expression of osteoclast-specific genes including *TRAP*, *Ctsk*, and fusion-related molecules [12] (Figure 4). The simultaneous suppression of multiple RANKL-responsive signaling cascades by 8-E likely contributes to its potent inhibitory effect at low micromolar concentrations. Notably, the parent compound Xanthatin has been previously shown to possess anti-inflammatory properties [13,14]. Our novel findings extend these observations by demonstrating that 8-E, a structural analog of xanthatin, dampens RANKL-induced osteoclast differentiation, suggesting that sesquiterpene lactones from *Xanthium* species may represent a class of compounds with broad activity against pathological signaling in bone and inflammatory diseases.

The α,β-unsaturated carbonyl moiety characteristic of sesquiterpene lactones is known to undergo Michael addition with nucleophilic cysteine residues in target proteins [15]. By analogy with experimentally validated sesquiterpene lactone–protein interactions, this reactivity is proposed to contribute to the inhibition of RANKL-induced signaling observed in this study, as key components of NF-κB signaling and upstream regulators of MAPK pathways contain functionally important cysteine residues. However, direct evidence for cysteine modification by 8-E has not yet been established. Future studies employing activity-based protein profiling (ABPP) using iodoacetamide-alkyne probes, or cysteine-masking approaches such as N-ethylmaleimide competition, will be required to identify the molecular targets of 8-E and validate this proposed mechanism.

Current anti-resorptive therapies, including bisphosphonates and denosumab, are effective in reducing fracture risk but are associated with adverse effects, particularly with long-term use [16]. In this context, small-molecule inhibitors that modulate intracellular signaling pathways may represent alternative therapeutic strategies with distinct pharmacological profiles. Several natural products have demonstrated anti-osteoclastogenic activity in vitro, and the potency of 8-E observed here suggests it compares favorably within this class.

The present study was conducted using in vitro cell culture models. Available in vivo evidence for 8-E and structurally related sesquiterpene lactones supports potential translational relevance. Anti-tumor and anti-angiogenic activities of xanthatin-class compounds have been demonstrated in xenograft models [8], and parthenolide, a structurally analogous sesquiterpene lactone sharing an α,β-unsaturated carbonyl pharmacophore, has shown efficacy in murine models of inflammatory bone loss [17]. Whether 8-E recapitulates these effects in established in vivo models of pathological bone loss, such as ovariectomy-induced osteoporosis or LPS-induced osteolysis, remains to be determined and represents an important next step in evaluating its therapeutic potential.

This study has limitations inherent to its preliminary nature. Functional bone resorption assays, detailed analysis of downstream transcriptional regulation, and in vivo validation will be necessary to fully assess the therapeutic potential of 8-E. In summary, our findings identify 8-Epixanthatin as an inhibitor of RANKL-induced osteoclastogenesis through suppression of NF-κB and MAPK signaling, providing a basis for further investigation of sesquiterpene lactones as scaffolds for anti-resorptive drug development. 

## 4. Materials and Methods

### 4.1. Cell Culture and Osteoclast Differentiation

RAW264.7 murine macrophages were cultured in Dulbecco’s modified Eagle’s medium (DMEM) supplemented with 10% fetal bovine serum (FBS) and antibiotics at 37 °C with 5% CO_2_. Osteoclast differentiation was induced as described previously [18]. Cells were seeded at 3 × 10^3^ cells/well in 96-well plates and stimulated with recombinant murine receptor activator of nuclear factor kappa-B ligand (RANKL) (30 ng/mL) for 5 days with medium replacement every 2 days. 8-Epixanthatin (8-E) (HY-137974, MedChemExpress, Monmouth Junction, NJ, USA) was added at the start of RANKL stimulation and maintained throughout differentiation. Final dimethyl sulfoxide (DMSO) concentration was ≤0.1%.

### 4.2. Cell Viability Assay

RAW264.7 cells were treated with 8-E (0.1–20 μM) for 48 h, and viability was assessed using a Cell Counting Kit-8 (CCK-8) assay (Servicebio, Wuhan, China), based on the reduction of water-soluble tetrazolium-8 (WST-8) to a water-soluble formazan by cellular dehydrogenases. Results are expressed as a percentage relative to the vehicle control. 

### 4.3. TRAP Staining and Quantification

After 5 days of differentiation, cells were fixed and stained for tartrate-resistant acid phosphatase (TRAP) activity using a commercial kit (Servicebio, Wuhan, China). TRAP-positive multinucleated cells (≥3 nuclei) were counted as mature osteoclasts under a Keyence BZ-X810 microscope (Keyence, Osaka, Japan) (20× magnification). At least five wells per condition were analyzed as described previously [19].

### 4.4. Western Blot Analysis 

RAW264.7 cells were serum-starved, pretreated with 8-E (10 μM) or vehicle for 2 h, and stimulated with RANKL (50 ng/mL) for 15 min. Cell lysates were subjected to sodium dodecyl sulfate–polyacrylamide gel electrophoresis (SDS-PAGE) and immunoblotting using antibodies against phosphorylated and total p65, extracellular signal-regulated kinase 1/2 (ERK1/2), c-Jun N-terminal kinase (JNK), and p38, with glyceraldehyde-3-phosphate dehydrogenase (GAPDH) as loading control as described elsewhere [20]. Protein bands were visualized by enhanced chemiluminescence (ECL) and quantified using ImageJ (National Institutes of Health (NIH)), Bethesda, MD, USA; version 1.53). Phosphorylated proteins were normalized to corresponding total protein levels.

### 4.5. Quantitative Real-Time PCR Analysis 

Total RNA was isolated from RAW264.7 cells treated with RANKL (30 ng/mL) in the presence or absence of 8-E (2.5 μM) for 5 days. cDNA was synthesized from 1 μg RNA, and quantitative real-time reverse transcription polymerase chain reaction (qRT-PCR) was performed using SYBR Green chemistry on a LightCycler^®^ 96 system (Roche, Basel, Switzerland). Melt-curve analysis was conducted to confirm single-product amplification. Relative gene expression was calculated using the 2^−ΔΔCt^ method with *Gapdh* as the internal control. Primer sequences are provided in Supplementary Table S1.

### 4.6. Statistical Analysis 

Data are presented as mean ± SD from at least three independent experiments. Statistical significance between two groups was determined using an unpaired Student’s *t*-test, while comparisons among multiple groups were performed using one-way analysis of variance (ANOVA) followed by Tukey’s multiple comparisons test (GraphPad Prism 9). A value of *p* < 0.05 was considered statistically significant. 

## Data Availability

The data that support the findings of this study are available from the corresponding author upon reasonable request.

## Supplementary Materials

The following supporting information can be downloaded at: https://www.mdpi.com/article/10.3390/ijms27083578/s1.
