# Supplementary material for: 8-Epixanthatin Suppresses RANKL-Induced Osteoclast Differentiation via Inhibition of NF-κB and MAPK Signaling
Source: Int J Mol Sci. 2026 Apr 17;27(8):3578. doi: 10.3390/ijms27083578 (PMC13115677; doi:10.3390/ijms27083578)
Supplement: Supplementary file 1 [file ijms-27-03578-s001.zip › ijms-4215925-supplementary.pdf]

Supplementary Materials

Table S1. Primer sequences used for quantitative RT-PCR analysis. All primers are presented in the 5′ to 3′ direction.

Gene	Gene Symbol	NCBI Gene ID	Forward Primer (5′→3′)	Reverse Primer (5′→3′)	Product Length (bp)
Tartrate-resistant acid phosphatase	*Acp5* (TRAP)	11433	CACTCCCACCCTGA-GATTTGT	CATCGTCTGCAC-GGTTCTG	118
Cathepsin K	*Ctsk*	13038	GAAGAAGACTCAC-CAGAAGCAG	TCCAGGTTATGGG-CAGAGATT	102
ATPase H^+ transport-ing V0 subunit D2	*Atp6v0d2*	242341	CAGAGCTG-TACTTCAATGTGGAC	AGGTCTCACACTG-CACTAGGT	111
Glyceraldehyde-3-phosphate dehydro-genase	*Gapdh*	14433	AGGTCGGTGTGAAC-GGATTTG	TGTAGACCATGTAGTT-GAGGTCA	123
